# Unraveling TEOSINTE BRANCHED1/CYCLOIDEA/PROLIFERATING CELL FACTOR Transcription Factors in Safflower: A Blueprint for Stress Resilience and Metabolic Regulation

**DOI:** 10.3390/molecules30020254

**Published:** 2025-01-10

**Authors:** Lili Yu, Xintong Ma, Mingran Dai, Yue Chang, Nan Wang, Jian Zhang, Min Zhang, Na Yao, Abdul Wakeel Umar, Xiuming Liu

**Affiliations:** 1 Engineering Research Center of the Chinese Ministry of Education for Bioreactor and Pharmaceutical Development, College of Life Sciences, Jilin Agricultural University, Changchun 130118, China; 15640309810@163.com (L.Y.); mxt7142020@163.com (X.M.); 13614328105@163.com (M.D.); cy18391348615@163.com (Y.C.); nanw@jlau.edu.cn (N.W.); as1220@163.com (J.Z.); nayao@jlau.edu.cn (N.Y.); 2 Institute for Safflower Industry Research of Shihezi University/Pharmacy College of Shihezi University/Key Laborataty of Xinjiang Phytomedicine Resource and Utilization, Ministry of Education, Shihezi 832003, China; 3 Monitoring and Testing Center for Ginseng and Antler Products, Ministry of Agriculture and Rural Affairs, Jilin Agriculture University, Changchun 130118, China; minz@jlau.edu.cn; 4 BNU-HKUST Laboratory of Green Innovation, Advanced Institute of Natural Sciences, Beijing Normal University at Zhuhai (BNUZ), Zhuhai 519087, China

**Keywords:** flavonoid biosynthesis, gene expression profiling, molecular crop improvement, plant regulatory networks, safflower (*Carthamus tinctorius* L.), TCP transcription factors

## Abstract

Safflower (*Carthamus tinctorius* L.), a versatile medicinal and economic crop, harbors untapped genetic resources essential for stress resilience and metabolic regulation. The TEOSINTE BRANCHED1/CYCLOIDEA/PROLIFERATING CELL FACTOR (TCP) transcription factors, exclusive to plants, are pivotal in orchestrating growth, development, and stress responses, yet their roles in safflower remain unexplored. Here, we report the comprehensive identification and characterization of 26 safflower *TCP* genes (*CtTCPs*), categorized into Class I (PROLIFERATING CELL FACTOR, PCF) and Class II (CINCINNATA and TEOSINTE BRANCHED1/CYCLOIDEA, CIN and CYC/TB1) subfamilies. Comparative phylogenetics, conserved motif, and gene structure analyses revealed a high degree of evolutionary conservation and functional divergence within the gene family. Promoter analyses uncovered light-, hormone-, and stress-responsive cis-elements, underscoring their regulatory potential. Functional insights from qRT-PCR analyses demonstrated dynamic *CtTCP* expression under abiotic stresses, including abscisic acid (ABA), Methyl Jasmonate (MeJA), Cold, and ultraviolet radiation b (UV-B) treatments. Notably, ABA stress triggered a significant increase in flavonoid accumulation, correlated with the upregulation of key flavonoid biosynthesis genes and select *CtTCPs*. These findings illuminate the complex regulatory networks underlying safflower’s abiotic stress responses and secondary metabolism, offering a molecular framework to enhance crop resilience and metabolic engineering for sustainable agriculture

## 1. Introduction

Safflower (*Carthamus tinctorius* L.), a member of the Asteraceae family, holds dual significance as a traditional Chinese herbal medicine and a modern cash crop in China. Its seeds are a rich source of linoleic acid, widely utilized in food additives [1] and biodiesel production [2]. Safflower petals, abundant in flavonoids, have applications in dyes [3], cosmetics [4], and food additives [5]. Clinically, safflower is employed to treat dysmenorrhea [6], cardiovascular diseases [7,8], and osteoporosis [9]. Furthermore, discarded safflower materials can be repurposed into environmentally friendly composite packaging membranes [10]. Despite its agricultural and medicinal significance, the molecular mechanisms regulating safflower’s stress resilience and secondary metabolism remain underexplored, particularly regarding transcriptional regulation by *TCP* genes.

The *TCP* transcription factor family is named after TB1 (Teosinte Branched 1) from maize, CYC (CYCLOIDEA) from goldenrod, and PCF1/2 from rice (Oryza sativa) [11]. TCP proteins are characterized by a conserved basic helix-loop-helix (bHLH) domain of approximately 60 amino acids [12], which divides the family into two major groups: Class I and Class II. Class I TCP proteins, also known as PCF or TCP-P class, feature a bHLH domain shorter by four amino acids compared to Class II [13], with a general binding sequence of GTGGGNCC [14]. Class II TCP proteins are further divided into CYC/TB1 and CIN subgroups, with CIN found ubiquitously across plants and CYC/TB1 specific to angiosperms [15]. These proteins prefer a binding sequence of GTGGNCCC, as indicated by DNA-binding preference studies [15].

*TCP* transcription factors are central to numerous developmental and stress-responsive processes in plants. *TCP* transcription factors are involved in critical growth and developmental processes such as seed germination [16], flowering [17], floral organ development [18], axillary bud development [19], cell cycle regulation [20], and senescence [21]. Additionally, TCP genes show differential expression under abiotic stresses such as drought [22], cold [23], ABA [24], MeJA [25], and salt stress [26], underlining their diverse regulatory roles in coordinating plant responses to environmental challenges.

The regulatory complexity of *TCP* transcription factors is exemplified by their interaction with microRNAs, particularly miR319, which targets *TCP* genes and modulates their expression in response to environmental cues [27,28]. This dynamic interaction suggests that *TCPs* integrate diverse developmental and stress-related signals, forming intricate feedback loops to optimize plant performance under varying conditions [29,30]. Beyond stress adaptation, *TCP* transcription factors regulate flowering time and reproductive development, traits critical for maximizing crop yield. *TCPs* integrate various signaling pathways to fine-tune flowering, ensuring reproductive success even under adverse environmental conditions [17,31]. Understanding these mechanisms in safflower could provide valuable insights for optimizing seed production and yield [32,33]. Additionally, *TCP* genes influence leaf morphology and architecture, traits vital for enhancing photosynthetic efficiency and resource use [34,35]. Targeted manipulation of *TCP* gene expression in safflower has the potential to improve light capture and water-use efficiency, particularly under stress conditions [36,37].

Recent advancements in genome-wide sequencing technologies have facilitated the identification of *TCP* gene families in various plant species, including *Arabidopsis thaliana* [38], tobacco [39], begonia [40], cowpea [41], chili pepper [42], frangipani [43], hickory [44], and passion fruit [45]. However, their roles in safflower remain largely unexplored. Given safflower’s importance as a source of oil, bioactive compounds, and pharmaceuticals, understanding the regulatory mechanisms involving *TCPs* is crucial for enhancing its productivity and stress resilience.

This study addresses the critical need to elucidate the functions of *TCP* transcription factors in safflower, particularly their roles in stress resilience and metabolic regulation. By identifying 26 *TCP* genes (*CtTCPs*) from the safflower genome, we comprehensively analyzed their physicochemical properties, phylogenetic relationships, gene structures, and cis-regulatory elements. The expression patterns of *CtTCPs* under abiotic stresses, including Cold, UV-B, ABA, and MeJA treatments, were assessed alongside flavonoid content changes under ABA stress using High-Performance Liquid Chromatography (HPLC). We hypothesize that specific *CtTCP* genes play a crucial role in modulating the expression of stress-related genes and secondary metabolite biosynthesis pathways, thereby contributing to the plant’s ability to adapt to abiotic and biotic stresses. This integrative approach aims to uncover the molecular networks through which *TCPs* regulate flavonoid biosynthesis and abiotic stress responses, providing foundational insights into safflower biology. Ultimately, this research offers strategies for genetic improvement to enhance safflower’s productivity and resilience in the face of global agricultural challenges.

## 2. Results

### 2.1. Identification of TCP Gene Family Members in Safflower

To identify all members of the *TCP* gene family in the safflower genome, we performed a BLASTP search using known TCP protein sequences from model plants, including *Arabidopsis thaliana* and *Oryza sativa* (Supplemental Table S2). The candidate sequences were further verified through an HMMER search using the Pfam domain PF03634, confirming the presence of the conserved TCP structural domain. This analysis led to the identification of 26 *CtTCP* genes, which were annotated as *CtTCP1* to *CtTCP26* (Table 1).

The physicochemical properties of CtTCP proteins were analyzed using the Expasy online tool, revealing significant variations in amino acid length, molecular weight, and isoelectric point. The amino acid lengths ranged from 115 (CtTCP14) to 713 (CtTCP16) residues, and the relative molecular weights spanned from 13.26 to 78.70 kDa. The isoelectric points (pI) varied between 5.23 (CtTCP12) and 10.58 (CtTCP14), indicating that CtTCP proteins exhibit diverse biochemical properties. The grand average of hydropathicity (GRAVY) index analysis classified all CtTCP proteins as hydrophilic, suggesting their likely interactions with aqueous cellular environments. Among the proteins, CtTCP17 was predicted to be the most stable, with an instability index of 35.83.

Subcellular localization predictions revealed that most CtTCP proteins are localized in the nucleus, consistent with their role as transcription factors. Notably, CtTCP3 and CtTCP17 were predicted to localize to the chloroplast and cytosol, respectively, indicating potential additional roles beyond nuclear transcriptional regulation. These results provide a foundational understanding of the structural and biochemical properties of the *CtTCP* gene family, highlighting their potential functional diversity in safflower.

### 2.2. Phylogenetic Analysis of CtTCP Genes

**Figure 1:**
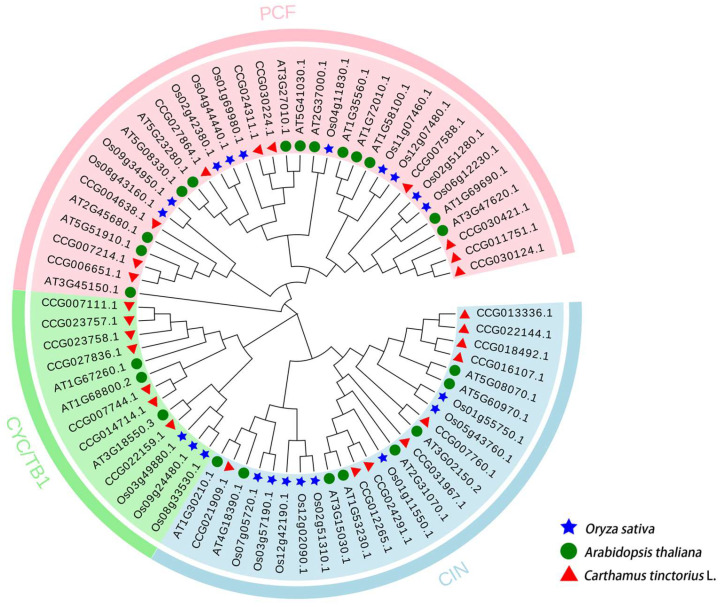
Phylogenetic analysis of *TCP* genes from *Carthamus tinctorius* L., *Arabidopsis thaliana*, and *Oryza sativa*. The figure displays a neighbor-joining (NJ) phylogenetic tree constructed using the full-length amino acid sequences of *TCP* genes from safflower (*Carthamus tinctorius* L., red triangles), *Arabidopsis thaliana* (green circles), and *Oryza sativa* (blue stars), generated with MEGAX software (version 10.1.8) and 1000 bootstrap replicates. The *TCP* gene family is grouped into three clades: PCF (pink), associated with cell cycle regulation; *CYC*/*TB1* (green), involved in axillary bud and leaf development; and CIN (blue), regulating organ size and shape. The 26 *CtTCP* genes clustered with 24 *TCP* genes from *Arabidopsis thaliana* and 21 from *Oryza sativa*, illustrating both conserved and species-specific functional diversification. This analysis provides insights into *TCP* gene evolution and specialization, offering a foundation for further functional studies.

TCP proteins are plant-specific transcription factors that play essential roles in plant growth, development, and abiotic stress response [46]. Therefore, phylogenetic trees of TCP proteins in *Oryza sativa*, *Arabidopsis thaliana*, and safflower were constructed (Figure 1) to study the evolutionary relationships between TCP proteins further. The CtTCP proteins were grouped into two classes (classes I and II) according to the differences in conserved domains. Ten *CtTCP* genes belonged to class I, the PCF subfamily. The remaining *CtTCP* genes belonged to class II, which can be further divided into two subfamilies: CYC/TB1 (seven members) and CIN (nine members). There were more members in Class II than in Class I, and in Class II, there were more members of the CIN subfamily than in the CYC/TB1 subfamily. These results were consistent with previous reports [11,47].

### 2.3. Motif and Gene Structure Analysis of CtTCPs

**Figure 2:**
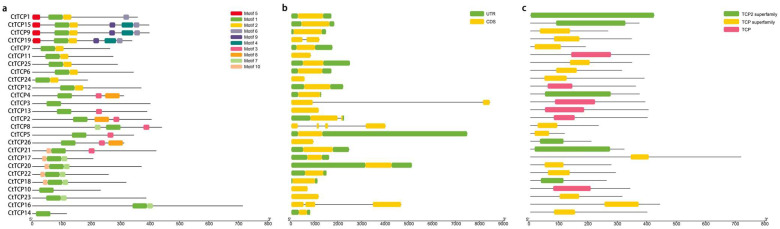
Protein motifs, gene structures, and conserved domains of *CtTCP* genes in safflower. This figure highlights the conserved motifs, gene structures, and conserved domains of the 26 *CtTCP* genes in safflower (*Carthamus tinctorius* L.). (**a**) Conserved motifs: The distribution of 10 conserved motifs is shown as colored blocks, indicating their length and position within the protein sequences. Distinct motif arrangements reflect the functional diversity and evolutionary relationships of CtTCP proteins. (**b**) Gene structures: The exon–intron organization is illustrated, with yellow boxes representing exons, green boxes for untranslated regions (UTRs), and black lines for introns. The variable exon and intron lengths indicate structural diversity, contributing to functional specialization. (**c**) Conserved domains: The green and yellow boxes represent subfamily-specific TCP domains, while the pink boxes indicate the core TCP domain. These positions validate the classification and conserved functionality of *CtTCP* genes. This analysis provides a foundation for understanding the structural and functional roles of *CtTCP* genes in safflower growth, development, and stress responses.

To gain further insight into the diversity of safflower *TCP* genes and their evolutionary relationships, a comparative analysis of conserved motifs within *CtTCP* genes was performed using the MEME website [48]. The analysis revealed ten conserved motifs within the 26 *CtTCP* genes examined (Figure 2a). The presence of Motif 1 in all 26 *CtTCP* genes suggests that it is highly conserved and likely plays a crucial role within the *CtTCP* gene family. In contrast, all members of the PCF subfamily contain Motif 2, while most CIN subfamily members possess Motif 7 and Motif 10. Motif 3 and Motif 8 are found in most CYC/TB1 members. These structural characteristics reflect functional differences between the subfamilies and correspond to their phylogenetic relationships. Thus, these conserved motifs further prove the taxonomic and functional diversity among safflower *TCP* gene subfamilies.

Furthermore, an examination of the structural domains of safflower TCP family members showed that all CtTCP proteins contain the conserved structural domains characteristic of the TCP family (Figure 2c). To delve deeper into the structural diversity of the *CtTCP* gene, an investigation of the introns and exons within the *CtTCP* gene was conducted (Figure 2b). The results indicated variability in the number of introns, ranging from 0 to 3. Specifically, *CtTCP2*, *CtTCP3*, *CtTCP8*, *CtTCP16*, and *CtTCP19* contained introns, while the other genes lacked them. The number of exons in *CtTCP* genes varies from one to four. Most *CtTCP* members contain a single exon, whereas *CtTCP8* has four exons, *CtTCP16* has three exons, and *CtTCP2*, *CtTCP3*, and *CtTCP19* each have two exons.

### 2.4. Cis-Acting Element Analysis of CtTCP Genes

**Figure 3:**
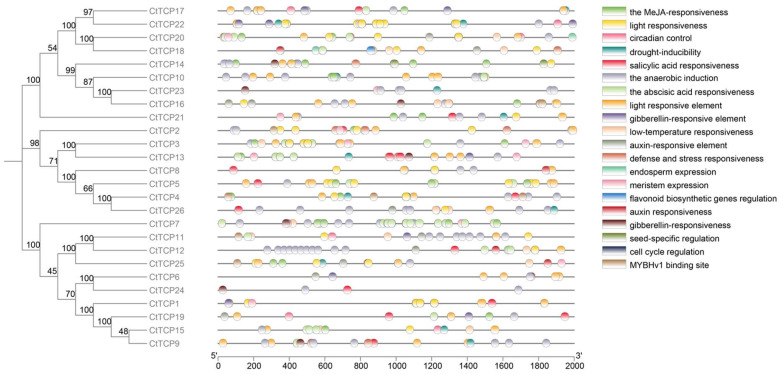
*Cis*-acting element analysis of *CtTCP* gene promoters in safflower. This figure shows cis-acting elements identified within the 2000 bp promoter regions of 26 *CtTCP* genes in safflower (*Carthamus tinctorius* L.) using PlantCARE. The phylogenetic tree on the left, constructed via neighbor-joining analysis with bootstrap values at key nodes, illustrates the evolutionary relationships among *CtTCP* genes. Promoter elements are displayed as colored blocks along the sequences, mapped from the 5′ to 3′ direction. The cis-acting elements are categorized as follows: (1) Hormone-responsive elements linked to gibberellin, abscisic acid, salicylic acid, auxin, and methyl jasmonate (MeJA). (2) Stress-responsive elements associated with drought, low temperature, defense, and anaerobic induction. (3) Growth- and development-related elements, including light responsiveness, circadian control, meristem expression, seed-specific regulation, and cell cycle regulation. (4) Regulatory pathway elements include flavonoid biosynthesis and MYBHv1 binding sites. The diverse distribution of these elements highlights the regulatory complexity of *CtTCP* genes and their roles in integrating hormonal, stress, and developmental signals, providing a basis for understanding their functions in safflower adaptation and growth.

To investigate the function of genes and how their expression is regulated, we conducted a *cis*-acting element analysis on the promoter sequences of *CtTCPs* using the Plant CARE online analysis software. The analysis revealed that *CtTCPs* contained 599 *cis*-acting elements, primarily comprising hormone-responsive and stress-responsive elements (Figure 3). The identified hormone-responsive elements included those responsive to gibberellin, salicylic acid, methyl jasmonate, abscisic acid, and growth hormone. The stress-responsive elements encompassed those responsive to drought, light, and low temperatures. Among all the response elements associated with *CtTCPs’* genes, the highest number was of light-responsive elements, totaling 194, which accounts for 32.4% of all cis-responsive elements. This was followed by abscisic acid-responsive elements, constituting 20% of the total cis-elements.

In contrast, elements that regulate circadian rhythms accounted for the smallest share, representing only 1% (Supplemental Table S3). Additionally, of the 26 *CtTCPs* studied, 17 were found to be responsive to MeJA, 13 were involved in the salicylic acid response, 14 responded to gibberellin, and 11 reacted to drought and low-temperature stress. Therefore, safflower *TCP* genes are hypothesized to play significant roles in regulating photoperiod, hormones, and abiotic stress in safflower. This provides a theoretical basis for future studies.

### 2.5. CtTCP Gene Expression Across Organs and Developmental Stages

**Figure 4:**
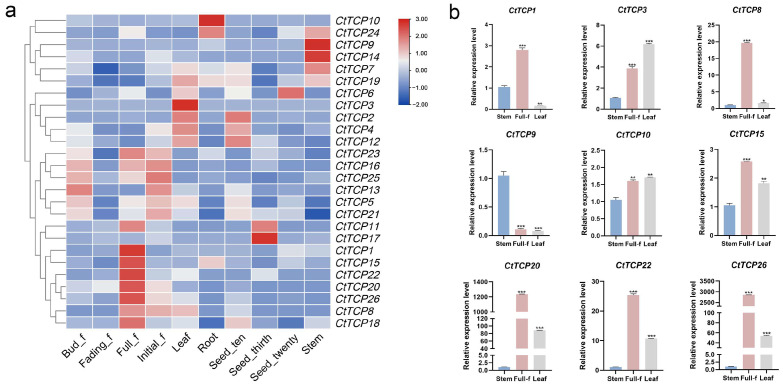
Expression profiles of *CtTCP* genes across different organs and developmental stages in safflower. This figure shows the differential expression patterns of *CtTCP* genes in various organs and developmental stages. (**a**) Heatmap: The expression levels of 26 *CtTCP* genes are displayed across roots, stems, leaves, flowers, and seeds at different developmental stages (bud, initial flowering, full bloom, fading flower, and seeds at 10, 20, and 30 days post-anthesis). The color scale (blue to red) indicates low to high expression. Distinct patterns include *CtTCP10* and *CtTCP24* with high expression in roots, *CtTCP7*, *CtTCP14*, and *CtTCP15* in stems, *CtTCP6* and *CtTCP17* in seeds, *CtTCP3* and *CtTCP12* in leaves, and *CtTCP22* and *CtTCP20* in flowers at full bloom. (**b**) qRT-PCR validation: The expression of nine *CtTCP* genes (*CtTCP1*, *CtTCP3*, *CtTCP8*, *CtTCP9*, *CtTCP10*, *CtTCP15*, *CtTCP20*, *CtTCP22*, *CtTCP26*) in stems, leaves, and flowers was consistent with transcriptome data. Statistical significance is shown by asterisks (* *p* < 0.05, ** *p* < 0.01, *** *p* < 0.001). This analysis highlights the diverse roles of *CtTCP* genes in safflower growth and development.

To explore the organs-specific and developmental stage-related expression profiles of *CtTCP* genes in safflower (*Carthamus tinctorius* ‘Jihong No.1’), transcriptome data from roots, stems, leaves, various flowering stages, and seed development periods were analyzed. The expression patterns are illustrated in Figure 4a. Among the identified genes, *CtTCP10* and *CtTCP24* exhibited the highest expression levels in roots, while *CtTCP7*, *CtTCP14*, *CtTCP15*, *CtTCP1*, and *CtTCP9* were predominantly expressed in stems. In seeds, *CtTCP6*, *CtTCP17*, and *CtTCP11* showed the highest expression levels, whereas *CtTCP3*, *CtTCP2*, *CtTCP4*, and *CtTCP12* were most abundantly expressed in leaves. Notably, *CtTCP22*, *CtTCP20*, *CtTCP26*, *CtTCP8*, and *CtTCP18* displayed peak expression in petals during full bloom.

To validate the transcriptome results, nine genes (*CtTCP1*, *CtTCP3*, *CtTCP8*, *CtTCP9*, *CtTCP10*, *CtTCP15*, *CtTCP20*, *CtTCP22*, and *CtTCP26*) were selected for RT-qPCR analysis. As shown in Figure 4b, the expression trends observed in stems, leaves, and flowers were consistent with the transcriptome data, confirming the reliability and accuracy of the transcriptomic analysis. This comprehensive expression profiling provides valuable insights into the functional roles of *CtTCP* genes in safflower organs’ development and flowering processes.

### 2.6. CtTCP Gene Expression Under Abiotic Stresses

**Figure 5:**
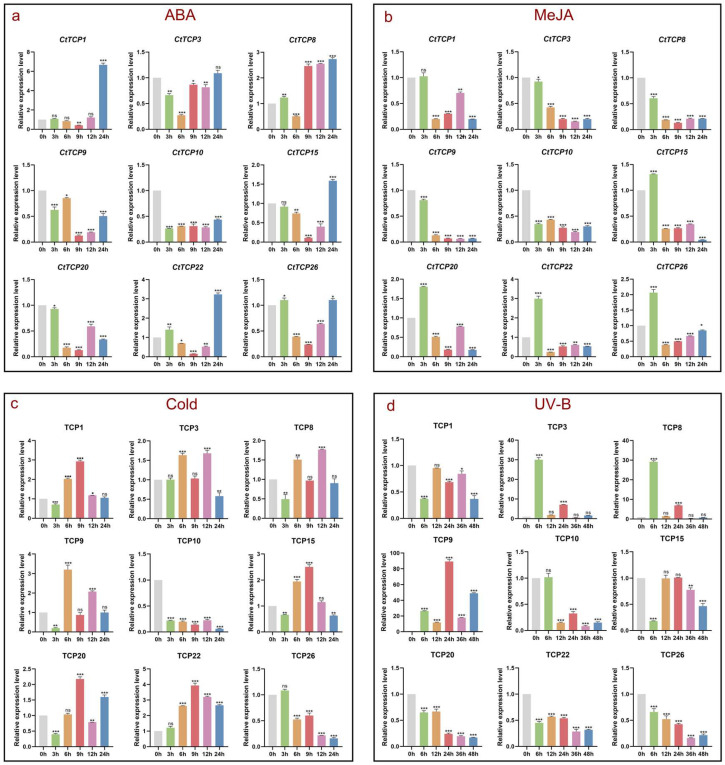
Expression profiles of *CtTCP* genes under abiotic stress treatments. The relative expression patterns of nine *CtTCP* genes were analyzed under four abiotic stress conditions using qRT-PCR, with results presented as mean ± standard deviation (SD). Significant differences compared to the control group (0 h) are indicated by asterisks (ns is not significant, * *p* < 0.05, ** *p* < 0.01, *** *p* < 0.001). (**a**) ABA (200 μmol): *CtTCP1*, *CtTCP8*, *CtTCP15*, and *CtTCP22* were upregulated, peaking at 24 h, while *CtTCP9*, *CtTCP10*, and *CtTCP20* showed a gradual decline. (**b**) MeJA (200 μmol): Most genes, including *CtTCP1*, *CtTCP3*, *CtTCP8*, *CtTCP9*, and *CtTCP10*, were consistently downregulated across all time points. (**c**) Cold (4 °C): *CtTCP10* and *CtTCP26* were downregulated, whereas *CtTCP1*, *CtTCP15*, *CtTCP20*, and *CtTCP22* peaked at 9 h, *CtTCP3* and *CtTCP8* at 12 h, and *CtTCP9* at 6 h. (**d**) UV-B (20,000 lx): *CtTCP10*, *CtTCP20*, *CtTCP22*, and *CtTCP26* were downregulated, with modest changes observed in other genes. This analysis underscores the stress-specific expression patterns of *CtTCP* genes and their potential roles in safflower’s abiotic stress responses.

Through Figure 3, we identified multiple cis-acting elements of the safflower *TCP* gene family, including hormone-responsive elements such as ABA and MeJA, light-responsive elements, and low-temperature-responsive elements. To investigate the involvement of *CtTCP* genes in abiotic stress responses, four stress conditions, ABA (200 μmol), MeJA (200 μmol), Cold (4 °C), and UV-B (20,000 lx), were applied to safflower. The expression levels of nine selected *CtTCP* genes (*CtTCP1*, *CtTCP3*, *CtTCP8*, *CtTCP9*, *CtTCP10*, *CtTCP15*, *CtTCP20*, *CtTCP22*, and *CtTCP26*) were analyzed using qRT-PCR, and the results are shown in Figure 5. These genes were selected based on results in Figure 4.

(a) ABA treatment: *CtTCP1*, *CtTCP8*, *CtTCP15*, and *CtTCP22* increased progressively during ABA treatment, with maximum expression observed at 24 h, indicating these genes are strongly induced by ABA signaling. In contrast, *CtTCP9*, *CtTCP10*, and *CtTCP20* showed gradual reductions in expression over time, suggesting they are repressed by ABA. This differential response may reflect functional specialization among *CtTCP* genes, where specific genes act as positive regulators of ABA-mediated stress pathways. In contrast, others act as negative regulators to maintain hormonal homeostasis.

(b) MeJA treatment: MeJA treatment resulted in a consistent downregulation of *CtTCP1*, *CtTCP3*, *CtTCP8*, *CtTCP9*, and *CtTCP10* over time, suggesting their potential negative involvement in MeJA-mediated stress responses. While the promoter regions of CtTCP1 and CtTCP8 do not contain MeJA-responsive cis-acting elements, the downregulation of these genes may be mediated through cross-talk between MeJA and ABA signaling pathways, highlighting the complex regulatory mechanisms underlying stress responses.

(c) Cold treatment: Cold stress-induced complex expression dynamics among *CtTCP* genes. While *CtTCP10* and *CtTCP26* exhibited steady declines in expression, reaching their lowest levels at 24 h, other genes, including *CtTCP1*, *CtTCP3*, *CtTCP8*, *CtTCP9*, *CtTCP15*, *CtTCP20*, and *CtTCP22*, were significantly upregulated. Among the upregulated genes, the expression of *CtTCP1*, *CtTCP15*, *CtTCP20*, and *CtTCP22* peaked at 9 h, suggesting an early response role in cold adaptation. Meanwhile, *CtTCP3* and *CtTCP8* reached their highest expression at 12 h, potentially representing a mid-term response, whereas *CtTCP9* peaked at 6 h, indicating its involvement in immediate cold stress responses. These time-dependent expression patterns reflect the complex regulatory roles of *CtTCP* genes in orchestrating a phased response to cold stress involving early, intermediate, and late-stage gene activation.

(d) UV-B treatment: UV-B stress led to a gradual reduction in the expression of *CtTCP10*, *CtTCP20*, *CtTCP22*, and *CtTCP26*, suggesting that these genes may play a repressive or non-essential role under UV-B exposure. In contrast, the expression levels of other *CtTCP* genes remained relatively stable, indicating their resistance to UV-B-induced suppression or potential involvement in maintaining baseline functions under UV-B stress.

The observed expression patterns across all stress treatments reveal that *CtTCP* genes exhibit distinct and stress-specific expression dynamics. Some genes, such as *CtTCP1*, *CtTCP8*, *CtTCP15*, and *CtTCP22*, appear to be key players in ABA and cold responses, showing significant upregulation. Conversely, genes like *CtTCP10* and *CtTCP26* showed consistent downregulation under cold and UV-B treatments, suggesting a potential inhibitory role under these conditions. Additionally, the shared trends among genes within the same subfamilies highlight conserved regulatory mechanisms, indicating that members of the same clade may have overlapping functions in stress adaptation.

This comprehensive analysis underscores the importance of *CtTCP* genes in abiotic stress tolerance. It provides insights into their potential as targets for genetic engineering to enhance safflower’s resilience to environmental stresses. Further functional studies are needed to validate these roles and explore their mechanistic contributions to stress response pathways.

### 2.7. ABA-Driven CtTCP Gene Links to Flavonoid Biosynthesis

**Figure 6:**
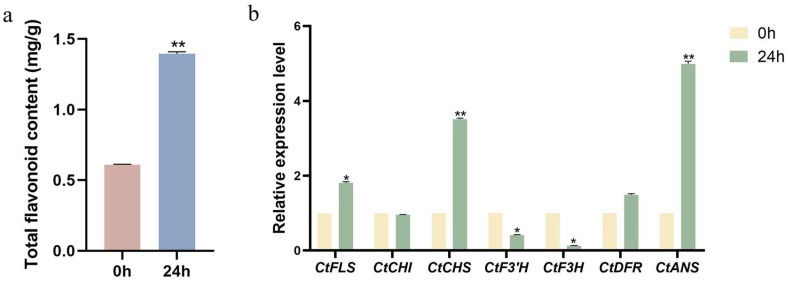
Effects of ABA treatment on flavonoid content and expression of key flavonoid biosynthetic genes in safflower. (**a**) Total flavonoid content: Flavonoid content (mg/g) in safflower leaves significantly increased ~2-fold after 24 h of ABA treatment (200 μmol) compared to the control (0 h). (**b**) Expression of key flavonoid biosynthetic genes: Relative expression levels of *CtFLS*, *CtCHI*, *CtCHS*, *CtF3’H*, *CtF3H*, *CtDFR*, and *CtANS* were analyzed after 24 h of ABA treatment. *CtFLS*, *CtCHS*, *CtDFR*, and *CtANS* were significantly upregulated, *CtF3’H* and *CtF3H* were downregulated, while *CtCHI* remained unchanged. Black asterisks denote significant differences compared to the control (* *p* < 0.05, ** *p* < 0.01), highlighting ABA’s regulatory role in flavonoid biosynthesis.

As illustrated in Figure 5b, most safflower *CtTCP* genes’ expression levels were significantly modulated after 24 h of treatment with 200 μmol ABA, suggesting their responsiveness to ABA-induced stress. To further investigate the connection between *CtTCP* gene expression and flavonoid biosynthesis under ABA stress, the total flavonoid content in safflower leaves was quantified after 24 h of ABA exposure, with untreated leaves as the control. As shown in Figure 6a, ABA treatment led to a marked ~2-fold increase in total flavonoid content compared to the control, indicating that ABA stress stimulates flavonoid accumulation in safflower.

To elucidate the molecular basis underlying this increase, the total RNA was extracted from leaves at 0 h and 24 h post-treatment, and the expression levels of key flavonoid biosynthetic genes were analyzed. As presented in Figure 6b, ABA treatment significantly upregulated the expression of *CtFLS*, *CtCHS*, *CtDFR*, and *CtANS*, which are involved in flavonoid biosynthesis. In contrast, *CtF3’H* and *CtF3H* were downregulated, while *CtCHI* showed no significant change. These expression profiles indicate that ABA stress selectively regulates the flavonoid biosynthetic pathway by activating specific enzymes while suppressing others.

Interestingly, the expression trends of these key biosynthetic genes closely aligned with the expression profiles of certain *CtTCP* genes. For example, *CtTCP1*, *CtTCP8*, *CtTCP15*, and *CtTCP22* were significantly upregulated after ABA treatment, mirroring the increased expression of *CtFLS*, *CtCHS*, *CtDFR*, and *CtANS*. Conversely, *CtTCP9*, *CtTCP10*, and *CtTCP20* showed reduced expression, corresponding to the downregulation of *CtF3’H* and *CtF3H*. This parallel between *CtTCP* gene expression and flavonoid biosynthetic gene regulation strongly suggests a functional link between these transcription factors and the modulation of flavonoid synthesis under ABA stress.

Taken together, these findings suggest that *CtTCP* transcription factors may interact with and regulate key flavonoid biosynthetic genes in response to ABA-induced stress. This regulatory network likely serves as a mechanism to enhance flavonoid production, which is known to play crucial roles in stress tolerance and metabolic adaptation. These results underscore the importance of *CtTCP* genes in coordinating metabolic responses to environmental stimuli, providing a foundation for further exploration of their regulatory roles in safflower.

## 3. Discussion

Transcription factors (TFs) play pivotal roles in regulating gene expression, mediating biological responses, and orchestrating developmental processes in plants and other organisms [49]. TCP transcription factors, a plant-specific TF family, have emerged as key regulators of plant development and stress responses [50]. Studies on TCP TFs have revealed their involvement in processes ranging from organ morphogenesis and cell cycle regulation to hormone-mediated stress responses [15,19]. In this study, we present the first comprehensive analysis of the *TCP* gene family in safflower (*Carthamus tinctorius* L.), a plant valued for its medicinal, industrial, and agricultural significance [5,6].

**Figure 7:**
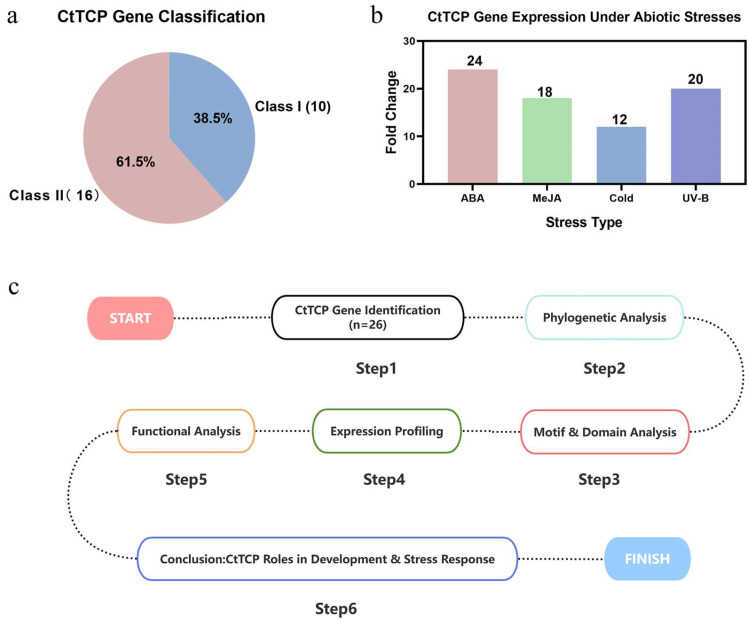
Comprehensive Conclusion of *CtTCP* Gene Roles in Safflower. This figure summarizes the key findings and conclusions of the study on *CtTCP* gene functions in safflower (*Carthamus tinctorius* L.). (**a**) *CtTCP* Gene Classification: A pie chart depicting the distribution of 26 *CtTCP* genes into two primary classes: Class I (10 genes, 38.5%) and Class II (16 genes, 61.5%), based on phylogenetic and structural analysis. Class II genes dominate, reflecting their evolutionary and functional diversification. (**b**) *CtTCP* Gene Expression Under Abiotic Stresses: A bar chart illustrating the relative fold change in *CtTCP* gene expression under four abiotic stress treatments: ABA (24-fold), MeJA (18-fold), Cold (12-fold), and UV-B (20-fold). These stressors elicited distinct gene expression responses, highlighting the regulatory roles of *CtTCP* genes in stress adaptation. (**c**) Workflow of the Study: A schematic representation of the methodological workflow and major conclusions. Key steps include: (1) *CtTCP* Gene Identification (n = 26): Comprehensive identification and annotation of *CtTCP* genes from the safflower genome. (2) Phylogenetic Analysis: Classification of *CtTCP* genes into Class I and Class II clades, supported by conserved domain analysis. (3) Motif and Domain Analysis: Detailed characterization of conserved motifs and structural domains within CtTCP proteins. (4) Expression Profiling: Analysis of *CtTCP* gene expression patterns across organs, developmental stages, and under abiotic stresses. (5) Functional Analysis: Validation of gene expression and functional roles in flavonoid biosynthesis and abiotic stress responses. (6) Conclusion: *CtTCP* genes exhibit multifaceted roles, including tissue-specific functions, regulation of flavonoid biosynthesis, and abiotic stress adaptation. Arrows indicate the progression of the study, emphasizing the integrative approach used to understand *CtTCP* gene functions. This figure encapsulates the study’s findings, highlighting the significance of *CtTCP* genes in safflower’s growth, development, and stress resilience.

We identified 26 *CtTCP* genes from the safflower genome, classifying them into Class I and Class II subfamilies based on conserved TCP domains (bHLH) (Table 1). While the ~1:2 ratio of Class I to Class II genes is consistent with findings in other plants, such as Arabidopsis [51], this ratio differs from certain other species like Prunus mume [52] (Figure 7a). Phylogenetic analysis revealed evolutionary conservation among *CtTCP* genes, with clear clustering into the PCF, CYC/TB1, and CIN subfamilies, mirroring results in other species like tobacco [39] and grapevine [53] (Figure 1). These evolutionary relationships suggest functional conservation within the TCP family, while species-specific expansions may indicate specialized roles in safflower adaptation and development.

The analysis of conserved motifs revealed a high degree of conservation across CtTCP proteins, with motif 1 representing the hallmark DNA-binding TCP domain (Figure 2a). Motif distribution patterns, highly conserved within subfamilies, imply that CtTCP proteins have subfamily-specific functional roles. These findings align with prior studies in crops like cotton, where motif organization is linked to TCP protein functionality [54]. Furthermore, exon–intron structural analysis revealed consistent patterns within subfamilies, supporting evolutionary conservation and functional specialization (Figure 2b). These conserved structures are particularly significant in elucidating the role of *CtTCP* genes as transcriptional regulators [31,55].

Cis-acting element analysis provided deeper insights into the potential regulatory roles of *CtTCP* genes (Figure 3). Promoters of *CtTCP* genes were enriched with hormone-responsive elements, including ABA, MeJA, gibberellin, and salicylic acid elements, as well as abiotic stress-related elements for drought and low temperature (Figure 3 and Figure 7b). These findings indicate that CtTCP genes integrate multiple signaling pathways, coordinating plant growth, development, and stress responses. Similar observations have been made in *TCP* gene families of other crops, such as pepper and cassava, where promoter elements facilitate hormone-stress crosstalk [23,42].

In this study, the expression profiles of *CtTCP* genes under abiotic stress (ABA, MeJA, cold, and UV-B) provided critical insights into their regulatory mechanisms. Under ABA treatment, *CtTCP1*, *CtTCP8*, *CtTCP15*, and *CtTCP22* exhibited significant upregulation, suggesting their roles as positive regulators of ABA signaling (Figure 5a). Interestingly, *CtTCP9*, *CtTCP10*, and *CtTCP20* displayed reduced expression, highlighting possible functional divergence within the family (Figure 5a). Similarly, *CtTCP20* and *CtTCP26* responded rapidly to MeJA, showing transient expression peaks that emphasize their roles in jasmonic acid-mediated stress responses (Figure 5b). These findings align with previous reports demonstrating the involvement of *TCP* genes in jasmonate signaling in plants like Artemisia annua and chrysanthemum [21,24].

The role of *CtTCP* genes in metabolic regulation was further underscored by their interaction with the flavonoid biosynthetic pathway under ABA stress. The 2-fold increase in total flavonoid content following ABA treatment (Figure 6a) suggests that *CtTCP* genes positively regulate secondary metabolism during stress. Enhanced expression of structural genes, including *CtFLS*, *CtCHS*, and *CtANS*, corroborates this hypothesis (Figure 6b). These findings indicate that *CtTCP1*, *CtTCP8*, *CtTCP15*, and *CtTCP22* may directly or indirectly influence flavonoid biosynthesis. Such regulatory roles have been observed in other species, where *TCP* genes modulate key metabolic pathways under stress [25]. Flavonoids, as essential secondary metabolites, not only contribute to plant stress tolerance but also have pharmaceutical significance, particularly in safflower, where flavonoids are used in cardiovascular therapies [6,8].

The response of *CtTCP* genes to UV-B and cold stress further highlights their versatility. Genes such as *CtTCP10*, *CtTCP20*, and *CtTCP26*, which were downregulated under UV-B, may act as negative regulators of UV-B-induced pathways, while others, like *CtTCP1* and *CtTCP15*, showed distinct patterns under cold stress, indicating their roles in cold acclimation (Figure 5c,d). These results are consistent with findings in other crops like Zea mays and Passiflora edulis, where *TCP* genes play integral roles in abiotic stress responses [45,56].

Overall (Figure 7c), the findings from this study significantly advance our understanding of *CtTCP* genes in safflower, emphasizing their multifunctionality in regulating growth, development, and stress responses. The observed integration of hormonal and environmental signals into secondary metabolic pathways highlights the potential of *CtTCP* genes as targets for genetic improvement.

## 4. Materials and Methods

### 4.1. Plant Materials and Stress Treatment

This study used the safflower (Jihong No.1) as experimental material. The seeds of Jihong No.1 were initially obtained from the Engineering Research Center of the Chinese Ministry of Education for Bioreactor and Pharmaceutical Development, Jilin Agricultural University. Safflower plants were grown in a greenhouse under a long-day condition (16 h light/8 h dark) at 25 °C with a relative humidity of 40%. The safflower samples were harvested at three plant organs: stem, leaf, and full flowering. In addition, three weeks of seedlings were treated with low-temperature processing (4 °C) [57], 200 μmol methyl jasmonate (Me-JA) [58], 200 μmol abscisic acid (ABA) [58], and UV-B (20000 lx) [59]. The leaves for each treatment were collected separately at 0, 3, 6, 9, 12, and 24 h after treatment. All samples were immediately frozen in liquid nitrogen and stored at −80 °C for further analysis.

### 4.2. Identification of the TCP Gene Family in Safflower

The safflower genome sequences were assembled and annotated, and the database was saved in our lab. The protein sequences of 24 *Arabidopsis* TCPs were downloaded from the *Arabidopsis* Information Resource (TAIR) database (https://www.arabidopsis.org/, accessed on 25 October 2024). NCBI BLASTP search (https://blast.ncbi.nlm.nih.gov/, accessed on 25 October 2024) was used to search and compare the amino acid sequence of the known *Arabidopsis* TCPs protein with that of safflower, and the candidate proteins of CtTCP were preliminarily screened. Then, the TCP conserved domain (PF03634) was subsequently obtained from the Pfam database (http://pfam.xfam.org/, accessed on 25 October 2024), and the Simple hidden Markov model (HMM) search tool in Tbtools(version 2.1) [60] was used to verify the presence of the conserved TCP domain in each candidate CtTCPs protein. To further identify the conserved domain of CtTCP proteins, all of the candidate proteins were examined using the Conserved Domain Database (CDD) (https://www.ncbi.nlm.nih.gov/Structure/cdd/wrpsb.cgi, accessed on 25 October 2024). We finally obtained 26 *CtTCP* genes after eliminating the non-TCP conserved domain and redundant sequences. Moreover, the features of 26 CtTCP proteins were predicted using the ExPaSy Proteomics Server (http://web.expasy.org/protparam/, accessed on 25 October 2024), and the prediction of the 26 CtTCP proteins subcellular localization was verified with the help of the WoLF-PSORT online webserver (http://wolfpsort.hgc.jp, accessed on 25 October 2024).

### 4.3. Phylogenetic Analysis of CtTCP Genes

The amino acid sequences of *Arabidopsis thaliana* TCP TFs were obtained from the TAIR database (https://www.arabidopsis.org/, accessed on 25 October 2024). Meanwhile, rice TCP protein sequences were obtained from the Plant TFDB database (https://planttfdb.gao-lab.org/index.php, accessed on 25 October 2024). Their full-length protein sequences and those of safflower were aligned with the Clustal X software (version 2.1). Then, a phylogenetic tree was constructed following the neighbor-joining (NJ) method in MEGA X software (version 10.1.8), with 1000 bootstrap replicates [61]. Subsequently, phylogenetic trees were drawn and visualized using the Evolview V.2 online website (https://www.evolgenius.info/evolview-v2/#login, accessed on 25 October 2024).

### 4.4. Analysis of TCPs Conserved Motifs and Gene Structure in Safflower

The conserved motifs of 26 CtTCP proteins were analyzed using the Multiple Em for Motif Elucidation (MEME, https://meme-suite.org/, accessed on 25 October 2024) online tool, and the maximum number of motifs was set to 10. The other parameters were kept as defaults [48]. The conserved domains of CtTCP TFs were analyzed using the NCBI conserved domain database (https://www.ncbi.nlm.nih.gov/Structure/cdd/wrpsb.cgi, accessed on 25 October 2024). The exon–intron structure of 26 *CtTCP* genes was analyzed using the Gene Structure Display Server 2.0 (GSDS) (http://gsds.cbi.pku.edu.cn/, accessed on 25 October 2024). Finally, the results of the above analyses were visualized using TBtools software (version 2.1).

### 4.5. Cis-Elements Analysis in CtTCP Promoters

The upstream 2000 bp sequence of *TCP* genes in the safflower genome database was extracted as the promoter to explore the *cis*-acting binding elements. The *cis*-acting elements of the 26 *CtTCP* genes in safflower were analyzed using the online tool PlantCARE (https://bioinformatics.psb.ugent.be/webtools/plantcare/html/, accessed on 25 October 2024), and the predicted results were visualized using the TBtools software (version 2.1).

### 4.6. RNA-Seq Expression Analysis

We retrieved raw RNA-Seq data from the safflower genome database to analyze the differential expression patterns of *CtTCP* genes across various organs and developmental stages, including root, stem, leaf, bud, initial flower, full bloom, fading flower, and seeds at 10-, 20-, and 30-days post-flowering. A value for fragments per kilobase of transcript per million fragments mapped (FPKM) was calculated for each gene, and the log2 (Fold Change) transformed values for each *CtTCP* family gene were used to generate a heatmap. A heat map of the expression profiles of *CtTCP* genes in different tissues and developmental stages was drawn using the TBtools software (version 2.1).

### 4.7. RNA Extraction and qRT-PCR

Total RNA was extracted from different tissues (stem, leaf, and full flowering) using RNAiso Plus reagent (TaKaRa, Beijing, China) according to the manufacturer’s protocol. One microgram (μg) of high-quality total RNA was used for the first-strand cDNA synthesis by HiFiScript All-in-one RT Master Mix for qPCR (CWBIO, Beijing, China). The qRT-PCR was performed with SuperStar Universal SYBR Master Mix (CWBIO, Beijing, China) using the Stratagene Mx3000P thermocycler (Agilent Technologies Inc., Santa Clara, CA, USA) system following the manufacturer’s instructions. The relative quantitative primers (Supplemental Table S1) were designed using the primer 5 software, and the *Ct*60S (KJ634810) gene was used as an internal control. Three biological replications of each sample were used for qRT–PCR analysis and the expression of nine selected genes in different tissues was calculated using the 2^−ΔΔCt^ method [62].

### 4.8. Measurement of Total Flavonoid Content

The fresh samples (0.1 g) were weighed, dried, and ground into a fine powder. Methanol (2 mL) was added to the powder, and ultrasonic extraction was performed at 50 °C for 1 h. After cooling, the extract was centrifuged at 12,000 rpm for 10 min, and the supernatant was filtered through a 0.22 μm filter membrane. The liquid chromatographic conditions were as follows: mobile phase consisting of 0.4% formic acid with a water-to-methanol ratio of 1:1, detection wavelength at 360 nm, and column temperature maintained at 25 °C. The analysis was conducted using a high-performance liquid chromatograph (HPLC) system (Agilent 1200) equipped with an Agilent ZORBAX 300SB-C18 (Agilent, Santa Clara, CA, USA) column (5 μm, 4.6 × 250 mm). Rutin standard was obtained from Solarbio (Beijing, China).

## 5. Conclusions

**Figure 8:**
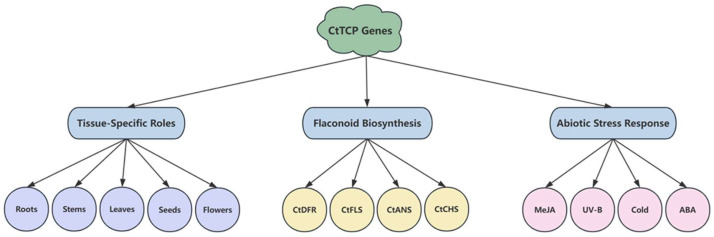
Hierarchical framework summarizing the roles of *CtTCP* genes in safflower growth, development, and stress responses. This figure illustrates the multifaceted roles of *CtTCP* genes in safflower (*Carthamus tinctorius* L.) based on tissue-specific expression, involvement in flavonoid biosynthesis, and abiotic stress responses. The central node represents the *CtTCP* gene family, with three major categories branching out: (1) Different Organs: *CtTCP* genes exhibit differential expression across various organs, including roots, stems, leaves, seeds, and flowers, emphasizing their diverse roles in tissue development and function. (2) Flavonoid Biosynthesis: *CtTCP* genes regulate key enzymes in the flavonoid biosynthetic pathway, such as CtFLS (Flavonol Synthase), CtCHS (Chalcone Synthase), CtDFR (Dihydroflavonol 4-Reductase), and CtANS (Anthocyanidin Synthase). These enzymes contribute to the biosynthesis of flavonoids, which play critical roles in plant defense and stress resilience. (3) Abiotic Stress Response: *CtTCP* genes respond to various abiotic stress conditions, including MeJA, ABA, cold, and UV-B. These responses highlight their regulatory role in stress signaling pathways and adaptation mechanisms. Nodes are color-coded to represent categories: gold for the central *CtTCP* genes, light blue for major functional categories, light green for tissues, light coral for flavonoid-related genes, and orange for abiotic stress responses. The hierarchical arrangement and directed edges depict the relationships and regulatory pathways involving *CtTCP* genes, providing a comprehensive overview of their functional dynamics in safflower.

In this study, we systematically identified and characterized 26 *CtTCP* genes in safflower, providing the first comprehensive analysis of this important transcription factor family in the species. The *CtTCP* genes were classified into Class I and Class II subfamilies, with phylogenetic and structural analyses confirming their evolutionary conservation. Cis-acting element analysis highlighted their potential roles in hormonal and stress-responsive signaling pathways. Expression profiling revealed that *CtTCP* genes exhibit organs-specific and stress-induced expression patterns, with distinct regulatory roles under ABA, MeJA, cold, and UV-B treatments (Figure 8). Notably, the observed upregulation of structural flavonoid genes and the corresponding increase in flavonoid content suggest that *CtTCP* genes integrate hormonal signals to enhance metabolic output, thereby improving stress tolerance. Overall, this study establishes a strong foundation for exploring the multifunctionality of TCP transcription factors in safflower, with implications for both basic research and practical applications in agriculture and medicine.

## Figures and Tables

**Table 1:** Information on 26 *TCP* gene family members in *Carthamus tinctorius* L.

Gene Name	Gene ID	Type	Protein Length	PI	MW (kDa)	Subcellular Localization	Instability Index	GRAVY
CtTCP1	CCG007588.1	PCF	356	7.97	37.43	Nucleus	44.27	−0.632
CtTCP2	CCG022159.1	CYC/TB1	403	6.79	45.73	Nucleus	47.7	−0.982
CtTCP3	CCG014714.1	CYC/TB1	399	9.47	44.92	Chloroplast	45.86	−0.548
CtTCP4	CCG023757.1	CYC/TB1	309	6.13	35.14	Nucleus	43.74	−0.754
CtTCP5	CCG023758.1	CYC/TB1	343	9.33	38.81	Nucleus	56.8	−0.74
CtTCP6	CCG024311.1	PCF	342	7.37	36.45	Nucleus	55.58	−0.768
CtTCP7	CCG027864.1	PCF	262	9.6	27.46	Nucleus	51.86	−0.461
CtTCP8	CCG027836.1	CYC/TB1	438	8.91	49.97	Nucleus	60.68	−0.957
CtTCP9	CCG030124.1	PCF	396	7.31	42.19	Nucleus	54.17	−0.581
CtTCP10	CCG031967.1	CIN	230	5.33	26.49	Nucleus	58.71	−0.886
CtTCP11	CCG006651.1	PCF	273	9.48	29.01	Nucleus	49.08	−0.418
CtTCP12	CCG007214.1	PCF	368	5.23	39.00	Nucleus	64.55	−0.393
CtTCP13	CCG007744.1	CYC/TB1	388	6.48	43.64	Nucleus	54.95	−1.032
CtTCP14	CCG007760.1	CIN	115	10.58	13.26	Nucleus	59.09	−0.538
CtTCP15	CCG011751.1	PCF	395	6.7	42.40	Nucleus	52.59	−0.698
CtTCP16	CCG012265.1	CIN	713	6.7	78.70	Nucleus	60.84	−0.927
CtTCP17	CCG013336.1	CIN	205	6.31	23.31	Cytosol	35.83	−0.745
CtTCP18	CCG016107.1	CIN	317	6.49	35.44	Nucleus	45.63	−0.902
CtTCP19	CCG030421.1	PCF	337	6.3	35.94	Nucleus	54.15	−0.64
CtTCP20	CCG018492.1	CIN	369	6.37	40.98	Nucleus	57.08	−0.9
CtTCP21	CCG021909.1	CIN	419	8.91	45.83	Nucleus	47.3	−0.829
CtTCP22	CCG022144.1	CIN	257	8.67	29.21	Nucleus	51.57	−0.669
CtTCP23	CCG024291.1	CIN	385	5.99	42.31	Nucleus	52.69	−0.927
CtTCP24	CCG030224.1	PCF	186	7.06	19.90	Nucleus	54.25	−0.579
CtTCP25	CCG004638.1	PCF	288	9.71	31.22	Nucleus	51.63	−0.487
CtTCP26	CCG007111.1	CYC/TB1	310	6.34	35.65	Nucleus	51.25	−0.94

## Data Availability

The sequencing data have been deposited into NCBI under the accession number PRJNA1160341. All data generated or analyzed during this study are included in the published article and Supplementary Files.

## Supplementary Materials

The following supporting information can be downloaded at: https://www.mdpi.com/article/10.3390/molecules30020254/s1. Table S1: qPCR primers; Table S2: Sequences of TCP family proteins from *Arabidopsis thaliana* and *Oryza sativa*; Table S3: Cis-acting elements of the *CtTCP* gene promoter in safflower.
